# Optic Nerve Sheath Ultrasound for the Detection and Monitoring of Raised Intracranial Pressure in Tuberculous Meningitis

**DOI:** 10.1093/cid/ciaa1823

**Published:** 2020-12-07

**Authors:** Joseph Donovan, Pham Kieu Nguyet Oanh, Nicholas Dobbs, Nguyen Hoan Phu, Ho Dang Trung Nghia, David Summers, Nguyen Thuy Thuong Thuong, Guy E Thwaites, Vietnam ICU Translational Applications Laboratory (VITAL) Investigators

**Affiliations:** 1 Oxford University Clinical Research Unit, Centre for Tropical Medicine, Ho Chi Minh City, Vietnam; 2 Centre for Tropical Medicine and Global Health, Nuffield Department of Medicine, University of Oxford, Oxford, United Kingdom; 3 Hospital for Tropical Diseases, Ho Chi Minh City, Vietnam; 4 Department of Clinical Neuroscience, Royal Infirmary of Edinburgh, Edinburgh, United Kingdom; 5 Vietnam National University School of Medicine, Ho Chi Minh City, Vietnam; 6 Pham Ngoc Thach University of Medicine, Ho Chi Minh City, Vietnam

**Keywords:** optic nerve sheath, ultrasound, tuberculous meningitis, intracranial pressure

## Abstract

**Background:**

Neurological complications of tuberculous meningitis (TBM) often lead to raised intracranial pressure (ICP) resulting in high morbidity and mortality. Measurement of optic nerve sheath diameter (ONSD) by point-of-care ultrasound may aid in the identification of raised ICP in TBM.

**Methods:**

From June 2017 to December 2019, 107 Vietnamese adults with TBM, enrolled in the ACT HIV or LAST ACT trials (NCT03092817, NCT03100786), underwent ONSD ultrasound at ≥1 of days 0, 3, 7, 14, 21, and day ±30 after enrollment. Demographic data, TBM severity grade, HIV coinfection status, and clinical endpoints by 3 months were recorded. ONSD values were correlated with disease severity, baseline brain imaging, cerebrospinal fluid parameters, and clinical endpoints.

**Results:**

267 ONSD ultrasound scans were performed in 107 participants over the first 30 days of treatment, with measurements from 0.38–0.74 cm. Paired baseline ONSD and brain imaging were performed in 63 participants. Higher baseline ONSD was associated with more severe disease and abnormal brain imaging (abnormal imaging 0.55 cm vs 0.50 cm normal imaging, *P* = .01). Baseline median ONSD was significantly higher in participants who died by 3 months (0.56 cm [15/72]) versus participants who survived by 3 months (0.52 cm [57/72]) (*P* = .02). Median ONSD was higher at all follow-up times in participants who died by 3 months.

**Conclusions:**

Higher ONSD was associated with increased disease severity, brain imaging abnormalities, and increased death by 3 months. ONSD ultrasound has a potential role as a noninvasive, affordable bedside tool for predicting brain pathology and death in TBM.

## Teaser

Neurological complications of tuberculous meningitis often elevate intracranial pressure. Increased optic nerve sheath diameter was associated with increased disease severity, brain imaging abnormalities, and increased death by 3 months. Optic nerve sheath diameter ultrasound may aid monitoring in tuberculous meningitis.

Tuberculous meningitis (TBM) accounts for 2–5% of all tuberculosis (TB) cases. Death results in up to 50% of those with TBM [1–4], largely due to severe neurological complications that are hard to predict and difficult to manage. Hydrocephalus, brain infarcts, and enlarging tuberculomas all contribute to the devastating morbidity and poor outcomes of TBM. These complications may cause raised intracranial pressure (ICP), which can quickly lead to coma and death. Early recognition and management of raised ICP are vital in order to minimize intracerebral damage and maintain cerebral perfusion.

Invasive intracranial monitoring is the gold standard for measuring ICP in brain injury. However, invasive monitoring, involving device insertion into the central nervous system (CNS), requires specialists in a neurocritical care setting. Invasive ICP monitoring is not available to most individuals with TBM, who primarily reside in settings with limited health resources. Use of minimally or noninvasive methods for raised ICP detection is common in brain infection (Table 1). Clinical assessment has limitations; monitoring performed after neurological deterioration may prove too late in TBM. Lumbar cerebrospinal fluid (CSF) opening pressure or counting CSF drops over time are often used as surrogate markers of ICP. However, pressure may not be evenly distributed throughout subarachnoid spaces, and few data support a positive correlation between lumbar CSF opening pressure and ICP [6, 7]. Fundoscopy is a low-cost technique used to visualize changes in the optic nerve head—papilloedema—that can indicate raised ICP. Yet, fundoscopy relies upon operator clinical skill and experience, and upon prior pupillary dilation to obtain satisfactory views. Importantly, papilloedema does not develop immediately after ICP elevation, limiting its use in acute monitoring.

**Table 1.** Methods for Detecting Raised Intracranial Pressure in Tuberculous Meningitis

Noninvasive	Invasive
Clinical assessment including GCS	Lumbar CSF opening pressure
Fundoscopy	Intraventricular catheters
ONSD ultrasound	Intraparenchymal pressure transducers
Transcranial Doppler ultrasound	Subarachnoid bolts
Brain imaging (CT or MRI)	Epidural transducers

Data from reference [5]. Abbreviations: CSF, cerebrospinal fluid; CT, computed tomography; GCS, Glasgow Coma Score; MRI, magnetic resonance imaging; ONSD, optic nerve sheath diameter.

**Figure 1.**
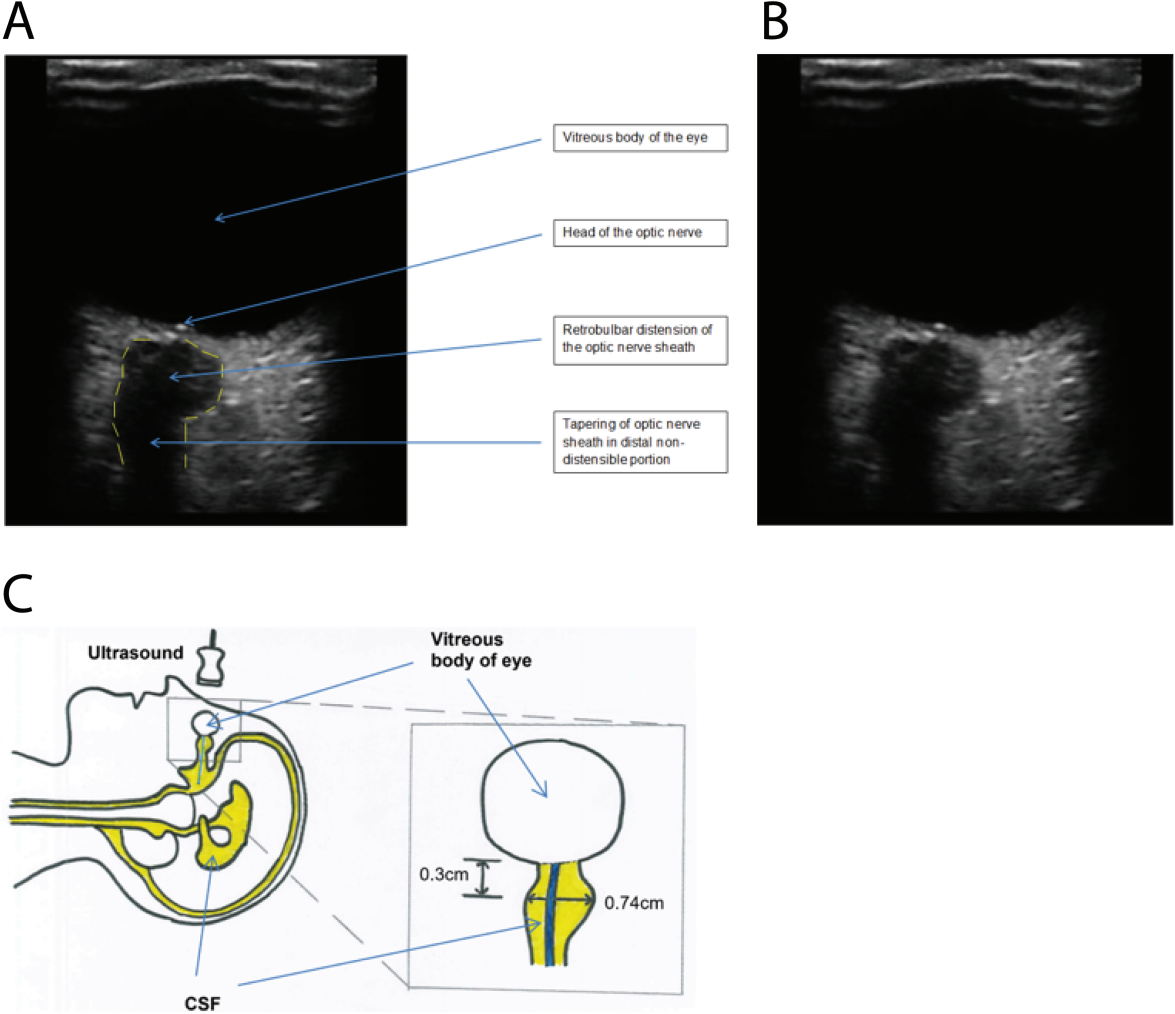
Distended optic nerve sheath consistent with raised intracranial pressure. Ultrasound images of the right eye are shown with (*A*) and without (*B*) descriptive labels. The borders of the optic nerve sheath are marked with a dotted line in panel *A*. In panel *C*, a diagram illustrates the appearances seen under ultrasound, showing how these appearances relate to patient position, CSF spaces (yellow) and optic nerve (blue). ONSD measured 0.3 cm from the posterior border of the globe of the eye was 0.74 cm. ONSD is measured as the distance inside the dura mater. Abbreviations: CSF, cerebrospinal fluid; ONSD, optic nerve sheath diameter.

Safe, cheap, and accessible monitoring, with high sensitivity for raised ICP detection, has the potential to enhance TBM management globally. The optic nerve, forming part of the CNS, is surrounded by a dural sheath that is distensible in its retrobulbar segment when ICP is elevated. Changes in optic nerve sheath diameter (ONSD) due to raised ICP occur in seconds [8]. Under ultrasound imaging the optic nerve appears hypoechogenic, surrounded by echogenic pia mater, hypoechogenic subarachnoid space, and then hyperechogenic dura mater and periorbital fat. A distended optic nerve sheath suggestive of elevated ICP is shown in Figure 1 [9]. Optic nerve sheath diameter ultrasound is safe, requires little training [10], and can be performed in less than 5 minutes. Studies in healthy individuals suggest that ONSD varies by ethnicity [11–16]; however, ONSD does not appear to vary with adult age, gender, or body mass index [15]; with waistline, head circumference, blood pressure, or pathological subtype [17]; or with side of eye measured [14]. Intraoperative and interoperative variabilities are acceptably low [15]. Meta-analyses confirm correlation between ONSD and invasively measured ICP [18–20], although studies of Europeans with noninfective brain pathology have predominated. Reports of ONSD ultrasound in brain infection are scarce, with only a single peer-reviewed publication in TBM [21]; 25 Indian adults with suspected TBM based on consistent magnetic resonance imaging (MRI) appearances (n = 25; mean ONSD, 5.81 mm) were compared with a control group where individuals lacked MRI appearances of TBM or papilloedema on fundoscopy (n = 120; upper limit of normal for ONSD, 4.37 mm) [21]. Larger studies are required to further investigate the role of ONSD ultrasound in TBM.

We sought to answer the following questions: Does elevated ONSD correlate with human immunodeficiency virus (HIV) status, TBM severity grade, or clinical endpoints by 3 months? Does elevated ONSD correlate with TBM brain-imaging abnormalities or features of raised ICP? Can an ONSD cutoff value predict TBM brain-imaging abnormalities or features of raised ICP? And how do ONSD values change during the first 30 days of anti-TB chemotherapy?

## METHODS

### Participants

We performed a prospective study of ONSD ultrasound in Vietnamese adults with TBM enrolled in the Adjunctive Corticosteroids for Tuberculous meningitis in HIV-infected adults (ACT HIV) [22] or Leukotriene A4 hydrolase Stratified Trial of Adjunctive Corticosteroids for HIV-uninfected adults with Tuberculous meningitis (LAST ACT) [23] clinical trials (Clinicaltrials.gov; NCT03092817 and NCT03100786, respectively). These 2 ongoing multicenter, randomized, placebo-controlled trials are assessing adjunctive corticosteroid therapy for the treatment of TBM in HIV coinfected adults and in HIV-uninfected adults stratified by leukotriene A4 hydrolase (*LTA4H*) genotype, respectively. Enrolled participants were 18 years or older, with clinical evidence of TBM based on consistent clinical and CSF findings, with or without HIV coinfection, and treated at the Hospital for Tropical Diseases (HTD) in Ho Chi Minh City, Vietnam. Patients were excluded if an additional brain infection to TBM was suspected, more than 6 consecutive days of anti-TB chemotherapy or systemic corticosteroids were received, or corticosteroids were mandatory or contraindicated. Informed consent was obtained from all participants or from a relative if the participant was incapacitated. Ethical approvals for ACT HIV and LAST ACT, which included ethical approval for ONSD ultrasound, were obtained from the Oxford Tropical Research Ethics Committee (36-16 and 52-16, respectively), the ethical committee of HTD (14/HDDD and 37/HDDD, respectively), and the Vietnam Ministry of Health (108/CN-BDGDD and 151/CN-BDGDD, respectively). Permission to publish these study data was obtained by the Chair of the respective Trial Steering Committees.

### Clinical Data

Demographic data (age, gender), baseline modified Medical Research Council (MRC) TBM severity grade [24], and HIV status were recorded. Final TBM diagnoses (definite, probable, possible) were assigned following the published uniform case definition for TBM (Supplementary Material 1) [25]. Death and new neurological complications by 3 months were recorded. Neurological complications were defined as a fall in Glasgow Coma Score (GCS) of 2 or more points for 48 hours or more, a focal neurological sign, seizure, cerebellar signs, coma, or cerebral herniation.

Baseline brain MRI (T1, T1 contrast, T1 contrast fluid attenuated inversion recovery, and T2-weighted sequences) was performed when safe to do so. Computed tomography (CT) imaging was performed when MRI was not possible. Hydrocephalus, number, and location of cerebral infarctions and tuberculomas; meningeal enhancement; and raised ICP features were recorded. Brain imaging was reported by an independent neuroradiologist, blinded to ONSD data, following a standard template (Supplementary Table 1).

For analysis, brain imaging was classified as abnormal if it contained 1 or more of the following: hydrocephalus, cerebral infarction(s), tuberculoma(s), meningeal enhancement, and/or features of raised ICP. Correlation between cross-sectional imaging findings and raised ICP is limited until it is particularly severe. However, a binary data point relating to imaging findings suggestive of raised ICP was recorded for each case. This was based on the global impression of the reporting neuroradiologist, taking into account the degree of sulcal effacement, severity of hydrocephalus, presence of transependymal edema, and any cerebral herniation.

### Optic Nerve Sheath Diameter Ultrasound Schedule and Procedure

Optic nerve sheath diameter ultrasound was performed on days 0, 3, 7, 14, 21, and day 30 (±1 day) after patient randomization into ACT HIV or LAST ACT, whenever possible. Day 30 ultrasound was performed only for inpatients. The ONSD ultrasound scans were performed by 1 of 2 clinicians with training in critical care ultrasound and experience in ONSD ultrasound. Preliminary data demonstrated both ultrasound operators met a pre-set acceptable interoperative variability of 0.3 mm; each operator performed the same 23 ONSD ultrasounds required to identify a 0.3-mm (or greater) difference if it existed, with a probability of 0.9.

Ultrasound was performed using Sonosite M-Turbo (Fujifilm Sonosite Ltd, Bothell, WA, USA) or Lumify (Philips, Amsterdam, Netherlands) ultrasound machines using a standard procedure. Briefly, the participant lay in bed, faced forward, looked forward, and closed their eyes. For unconscious patients, the head was gently turned to forward facing. An ultrasound probe with a small volume of ultrasound gel was placed gently over the temporal portion of the upper eyelid. No pressure was applied to the eye. An image was selected where the optic nerve sheath was viewed at its widest. Imaging quality was assessed by ensuring the optic nerve sheath was seen to within 1 mm of the globe of the eye, 6 mm of continuous optic nerve was seen, and there was absence of movement artefact. At 0.3 cm from the posterior border of the globe of the eye, the sheath diameter was measured. The ONSD measurements were recorded twice for each eye, and then an average of all 4 measurements was calculated.

### Treatment

All participants received anti-TB chemotherapy following Vietnamese national guidelines. Rifampicin, isoniazid, pyrazinamide, and ethambutol were given for at least the first 2 months of treatment if drug resistance was not suspected or proven. Pyrazinamide was then stopped, and rifampicin, isoniazid, and ethambutol were continued until 12 months of anti-TB chemotherapy was received in total. Drug doses and second line regimens are described in Supplementary Material 2. All participants were randomized to dexamethasone or placebo (“study drug”), a double-blinded allocation following a 1:1 randomization (except for *LTA4H* TT-genotype participants without HIV from LAST ACT [~7% total participants] who all received dexamethasone). The study drug was administered over 8 weeks (TBM severity grade 2 or 3) or 6 weeks (TBM severity grade 1), following a tapering course, with weekly reductions (Supplementary Table 2). The ACT HIV and LAST ACT trials are ongoing and treatment allocations remain blinded. Results for both trials are expected to be available in 2023.

### Statistical Analysis

Optic nerve sheath diameter was compared with brain imaging if both were performed within a 72-hour period. Analysis populations were created by separating study participants into groups based on the presence or absence of raised ICP, and on the presence or absence of abnormal appearances, on brain MRI/CT at baseline. Median ONSD was calculated for each group given nonnormal distribution of ONSD values. A sample-size calculation was performed based upon noninfection brain disease [21,26], given limited TBM data available for this purpose. Using a 5% significance level, 90% power to detect effect size, and an expected difference in means of 1.3 mm with a standard deviation of 1.1 mm, we calculated that 15 patients were required per group (abnormal brain imaging vs normal brain imaging) to reject the null hypothesis if it were false. Comparison between proportions was assessed by the chi-squared test. Non–normally distributed data were compared using the Wilcoxon rank-sum test. Correlation between continuous variables was performed using Spearman’s rank correlation coefficient. Data were analyzed using The R Foundation (version 3.6; R Project for Statistical Computing).

## RESULTS

### Study Population

From June 2017 to December 2019 inclusive, 107 Vietnamese adults with TBM had 267 ONSD ultrasound scans performed at day 0 (n = 72), day 3 (n = 48), day 7 (n = 45), day 14 (n = 44), day 21 (n = 42), and day 30 (n = 16). Four images were recorded at each of these 267 scanning time points. The median age of the study population was 37 (interquartile range, 29–45) years; 68.2% (73/107) of participants were male and 31.8% (34/107) were female. Final diagnoses of the study population were as follows: 75.7% (81/107) definite TBM, 12.1% (13/107) probable TBM, and 12.1% (13/107) possible TBM. Modified MRC TBM severity grades were as follows: grade 1, n = 33; grade 2, n = 58; grade 3, n = 16. Thirty-five of 107 (32.7%) participants had HIV coinfection.

### Baseline Optic Nerve Sheath Diameter Associations

Baseline ONSD was performed in 67.3% (72/107) of participants. Median baseline ONSD is shown by sex, final diagnosis, MRC TBM grade, and HIV coinfection status in Table 2. Baseline ONSD significantly increased with more severe disease (grade 1, 0.50 cm; grade 2, 0.55 cm; grade 3, 0.56 cm) (*P* = .01). Coinfection with HIV was not significantly associated with increased ONSD at baseline. No significant correlation was seen between baseline ONSD and baseline temperature, lumbar CSF opening pressure, CSF white blood cells, CSF lactate, CSF protein, or CSF/blood-glucose ratio (Supplementary Table 3).

**Table 2.** Median Baseline Optic Nerve Sheath Diameter by Subcategories

	Total (N = 72), n (%)	Median ONSD, cm	*P*
All patients	72	0.53	
Sex			
Male	47 (65.3)	0.52	.89
Female	25 (34.7)	0.54	
Final diagnosis			
Definite TBM	53 (73.6)	0.54	.25^a^
Probable TBM	7 (9.7)	0.52	
Possible TBM	12 (16.7)	0.51	
MRC TBM grade			
1	23 (31.9)	0.50	.01^b^
2	39 (54.2)	0.55	
3	10 (13.9)	0.56	
HIV status			
Positive	20 (27.8)	0.56	.17
Negative	52 (72.2)	0.52	

The Wilcoxon rank-sum test was used to compare ONSD values. “Total” reflects the total number of observations available for the corresponding variable. MRC grades are as follows: grade 1 indicates a GCS of 15 with no neurological signs, grade 2 a GCS of 11 to 14 (or 15 with focal neurological signs), and grade 3 a GCS of 10 or less. Abbreviations: GCS, Glasgow Coma Score; HIV, human immunodeficiency virus; MRC, Medical Research Council; ONSD, optic nerve sheath diameter; TB, tuberculosis; TBM, tuberculous meningitis.

^a^Definite TBM compared with nondefinite.

^b^Grade 1 compared with grades 2 and 3. Two final diagnoses scoring as “Not TBM” with the uniform case definition [25] were converted to “Possible TBM” on the basis that clinical diagnosis was of TBM, and anti-TB chemotherapy was received.

### Association Between Optic Nerve Sheath Diameter and Brain Imaging

We set out to investigate whether increased ONSD correlated with brain imaging consistent with raised ICP or with abnormal brain imaging appearances. There were 63 participants for whom ONSD and brain imaging were performed within 72 hours of each other at the start of treatment. In 6 of 63 participants (9.5%), brain imaging suggested raised ICP, and in 90.4% of participants (57/63) brain imaging did not suggest raised ICP. Median ONSD for the raised-ICP and non–raised-ICP groups were 0.55 cm and 0.52 cm, respectively (*P* = .59). In this same group of 63 participants, 39 of 63 (61.9%) had brain imaging with abnormal appearances consistent with TBM, and 38.1% of participants (24/63) had normal brain imaging. Median ONSDs for the abnormal imaging and normal imaging groups were 0.55 cm and 0.50 cm, respectively (*P* = .01). Median ONSD values by brain pathology groups were as follows: hydrocephalus, 0.55 cm (n = 10); tuberculoma(s), 0.52 cm (n = 13); cerebral infarction(s), 0.55 cm (n = 18); and meningeal enhancement, 0.52 cm (n = 29). To further investigate the difference in ONSD between normal and abnormal brain-imaging groups, TBM severity and CSF inflammatory parameters were compared between these 2 groups (Table 3). In the abnormal brain-imaging group there were significantly elevated CSF neutrophils and CSF lactate, and significantly reduced CSF- to-blood glucose ratio, consistent with increased disease severity in this group.

**Table 3.** Disease Severity and Cerebrospinal Fluid Inflammatory Parameters for Normal and Abnormal Brain-Imaging Groups

	Normal Brain Imaging (n = 24)		Abnormal Brain Imaging (N = 39)		
	Total No.	Values	Total No.	Values	*P*
Age, median (IQR), years	24	41 (28–50)	39	34 (29–40)	.08
Sex, n (%)	24		39		1.0
Male		16 (66.7)		25 (64.1)	
Female		8 (33.3)		14 (35.9)	
MRC TBM grade, n (%)	24		39		.09^a^
1		12 (50)		10 (25.6)	
2		11 (45.8)		25 (64.1)	
3		1 (4.2)		4 (10.3)	
HIV status, n (%)	24		39		1.0
Positive		6 (25)		9 (23.1)	
Negative		18 (75)		30 (76.9)	
Highest temperature, median (IQR), °C	24	38.7 (38.0–39.5)	39	39.0 (38.7–39.5)	.31
Lumbar CSF opening pressure, median (IQR) cm H_2_0	15	18 (15–24)	26	20 (17–29)	.54
CSF WBC count, median (IQR), cells/mm^3^	24	283 (137–501)	39	304 (200–538)	.32
CSF neutrophils, median (IQR), %	23	13 (11–30)	39	48 (20–73)	.002
CSF neutrophil count, median (IQR), cells/mm^3^	23	43 (19–120)	39	142 (39–229)	.01
CSF-to-blood glucose ratio, median (IQR)	24	0.39 (0.24–0.44)	39	0.27 (0.23–0.36)	.02
CSF lactate, median (IQR), mmol/L	24	4.0 (3.0–5.7)	39	5.8 (4.7–7.7)	.001

Abbreviations: CSF, cerebrospinal fluid; HIV, human immunodeficiency virus; IQR, interquartile range; MRC, Medical Research Council; TBM, tuberculous meningitis; WBC, white blood cell.

^a^Grade 1 compared with grades 2 and 3. The Wilcoxon rank-sum test and chi-squared test were used to compare averages of continuous and categorical data, respectively. Highest temperature, lumbar CSF opening pressure, CSF WBC count, CSF neutrophil percentage, CSF-to-blood glucose ratio, and CSF lactate are non–normally distributed and are shown as median (IQR).

### Response to Treatment and Outcomes by 3 Months

**Figure 2.**
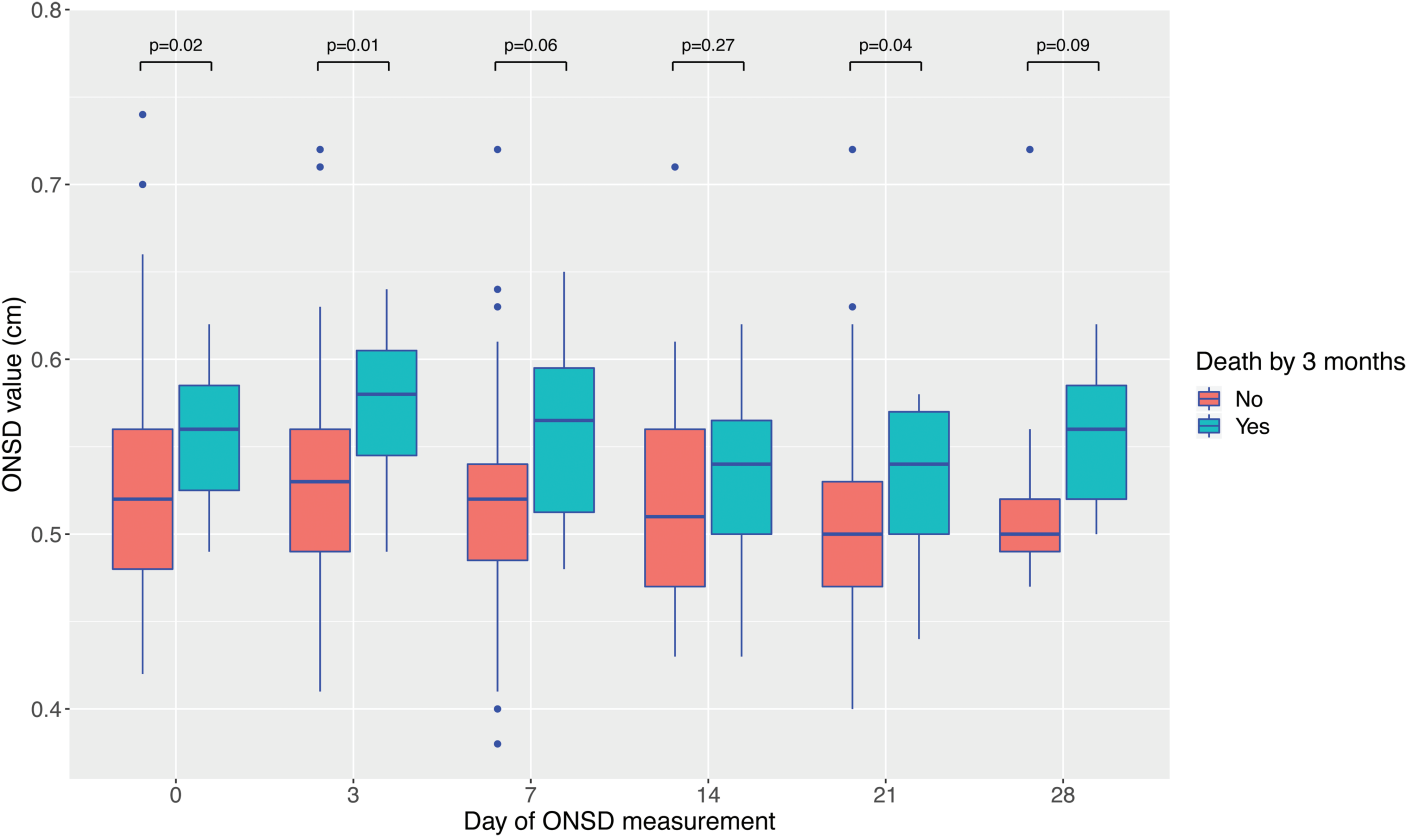
ONSD over 30 days of anti-TB chemotherapy, stratified by death by 3 months. For each individual boxplot, the central horizontal bar represents the median value. The box contains data between the third quartile (upper end of box) and the first quartile (lower end of box). Vertical lines above and below each box extend to the most extreme data point that is within 1.5× the vertical height of the box. Dots represent individual data points outside of these limits. *P* values represent statistical comparison of ONSD values performed by the Wilcoxon rank-sum test. Abbreviations: ONSD, optic nerve sheath diameter; TB, tuberculosis.

Baseline ONSD was significantly higher in participants who died (0.56 cm [15/72]) versus those who survived (0.52 cm [57/72]) (*P* = .02) (Table 4). In addition, median ONSD was higher in participants who died by 3 months versus those who survived at 3 months at all follow-up time points (days 3, 7, 14, 21, and 28) (Figure 2).

**Table 4.** Median Baseline Optic Nerve Sheath Diameter by Clinical Endpoints

	Total (N = 72), n (%)	Median ONSD, cm	*P*
Neurological complication by 3 months			
Yes	12 (16.7)	0.53	.61
No	60 (83.3)	0.53	
Death by 3 months			
Yes	15 (20.8)	0.56	.02
No	57 (79.2)	0.52	

*P* values represent comparison for ONSD values by the Wilcoxon rank-sum test. “Total” reflects the total number of observations available for the corresponding variable. MRC grades are as follows: Grade 1 indicates a GCS of 15 with no neurological signs, grade 2 a GCS of 11 to 14 (or 15 with focal neurological signs), and grade 3 a GCS of 10 or less. Abbreviations: GCS, Glasgow Coma Score; MRC, Medical Research Council; ONSD, optic nerve sheath diameter.

**Figure 3.**
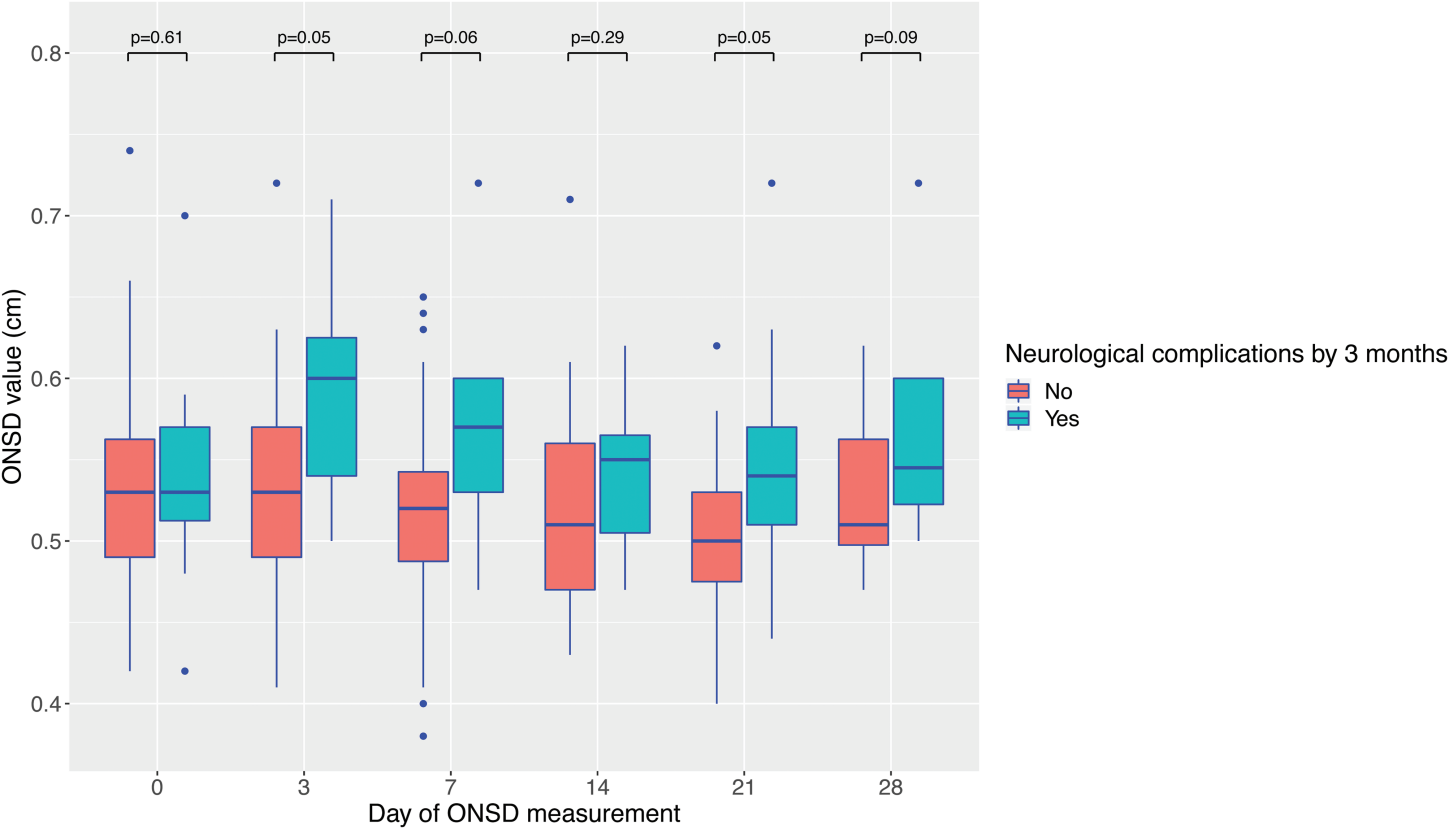
ONSD over 30 days of anti-TB chemotherapy, stratified by neurological complications by 3 months. For each individual boxplot, the central horizontal bar represents the median value. The box contains data between the third quartile (upper end of box) and the first quartile (lower end of box). Vertical lines above and below each box extend to the most extreme data point that is within 1.5× the vertical height of the box. Dots represent individual data points outside of these limits. *P* values represent statistical comparison of ONSD values performed by the Wilcoxon rank sum test. Abbreviations: ONSD, optic nerve sheath diameter; TB, tuberculosis.

There was no significant difference in baseline ONSD between participants who experienced neurological complications by 3 months versus participants who did not (0.53 cm vs 0.53 cm, respectively; *P* = .61). Follow-up data suggested a trend of higher ONSD in participants experiencing neurological events by 3 months versus participants without neurological events (Figure 3).

A receiver operating characteristic (ROC) curve was constructed to investigate whether ONSD could predict death by 3 months (Supplementary Figure 1), with acceptable sensitivity and specificity. A baseline ONSD of 0.53 cm or above predicted death by 3 months with 73% sensitivity and 54% specificity.

**Figure 4.**
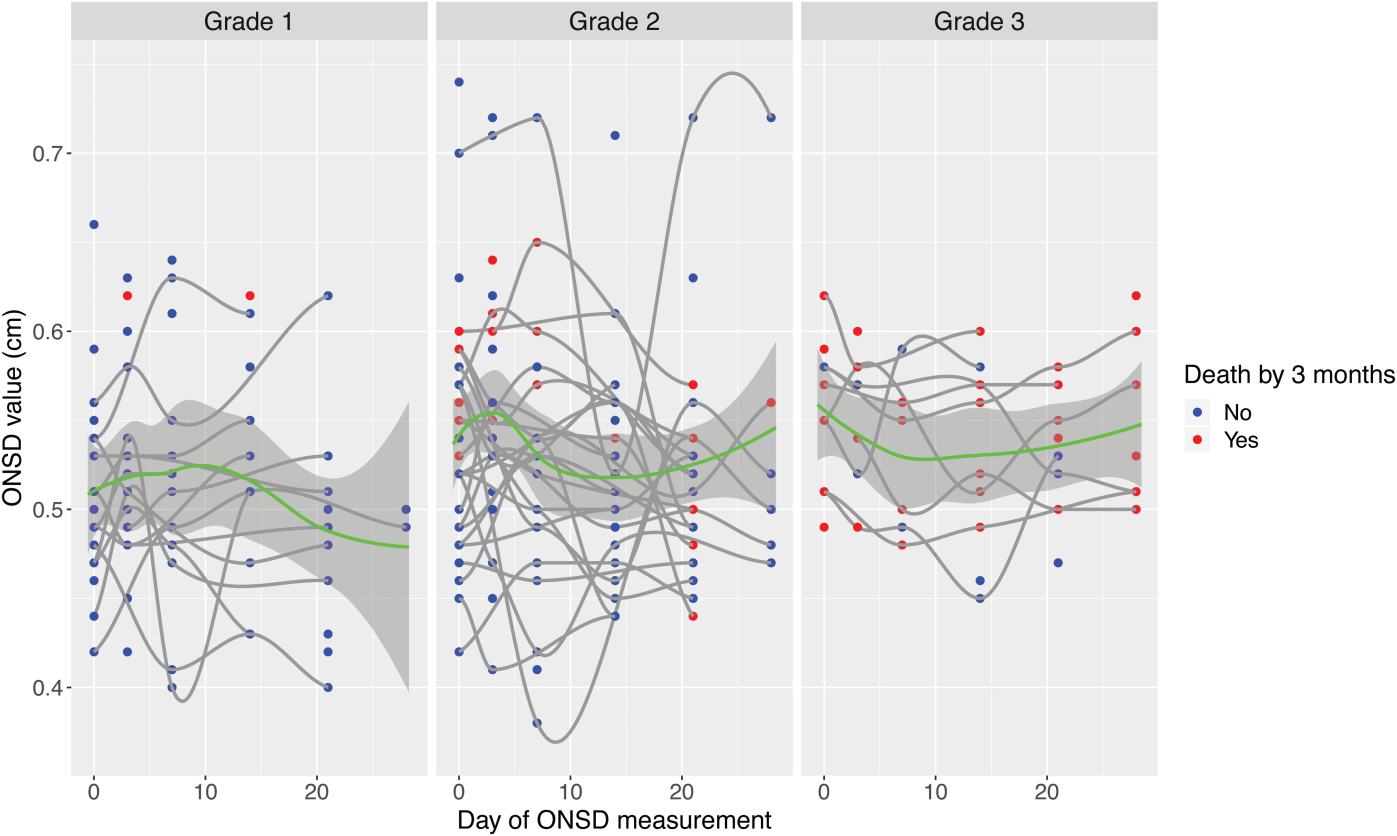
ONSD values over 30 days of anti-TB chemotherapy, stratified by TBM severity grade and death by 3 months. Individual data points represent individual ONSD values at a specified day of measurement. Data are shown stratified by grades 1, 2, and 3, where grade refers to MRC TBM severity grade. Blue dots represent ONSD values in patients who were alive by 3 months, whereas red dots represent ONSD values in patients who died by 3 months. The mean ONSD value across time points, for each grade, is represented by a green line with an associated 95% CI. Abbreviations: CI, confidence interval; MRC, Medical Research Council; ONSD, optic nerve sheath diameter; TB, tuberculosis; TBM, tuberculous meningitis.

Higher ONSD values were observed in those with more severe disease (Figure 4). In participants with grade 1 TBM, ONSD increases but then returns to baseline, consistent with ongoing recovery from TBM. In grade 2 and grade 3 TBM, ONSD continues to trend higher by 30 days, consistent with more severe disease in these groups.

## DISCUSSION

More evidence guiding the best detection and management of raised ICP in TBM is required. Point-of-care tools such as ONSD ultrasound may aid in increased ICP detection in TBM and guide management. In our study, ONSD was significantly higher in participants with abnormal brain imaging compared with participants with normal brain imaging. Baseline ONSD predicted death in the first 3 months of treatment.

Neurocomplications of TBM may elevate ICP. We showed that median ONSD was significantly higher in participants with brain imaging consistent with common TBM appearances, compared with that in participants with normal brain imaging. Comparison of CSF parameters between these brain-imaging groups showed significantly increased median CSF neutrophil percentage and lactate, and a significantly reduced CSF-to-glucose ratio, in the abnormal brain-imaging group versus the normal brain-imaging group. This more inflammatory CSF profile in the abnormal brain-imaging group reflects an increased disease severity. However, we were unable to directly associate baseline CSF inflammatory parameters with higher baseline ONSD.

Further, baseline ONSD was significantly higher in participants who died by 3 months than in those who did not. This suggests that ONSD is elevated by brain pathology that leads to worse clinical outcomes. This correlation between ONSD and severe TBM disease leading to poor outcomes illustrates the potential value of ONSD in the management of TBM. Previous data have correlated ONSD and other proxy markers of raised ICP [21]; however, our study is the first to associate higher ONSD with worse outcomes in TBM. In clinical practice, ONSD ultrasound therefore has the potential to enable earlier identification of neuroinflammatory complications that may progress to death, allowing prompt investigation and management. Using ROC curve analysis we identified a “best” ONSD cutoff value of 0.53 cm to separate participants who died by 3 months from those who survived by 3 months.

Our study has limitations. It was not possible to perform ONSD ultrasound at every time point for each participant, due to constraints on operator, participants, and resources. Most scans were performed by a single operator. While this allowed for consistency in scanning technique and reduced interoperator variability, it also meant that measurement could not be reviewed by a second operator. Optic nerve sheath diameter ultrasound itself has limitations, lacking standardization of technique and value interpretation. Additionally, ONSD appearances apparently consistent with raised ICP may in fact reflect nonraised ICP pathology. Solid optic nerve sheath thickening (eg, in ophthalmopathy of Graves’ disease or an optic nerve sheath meningioma) or severe orbital congestion (eg, due to an arteriovenous fistula) may produce confounding appearances [27]. However, these findings are rare.

A further limitation is that we compared ONSD and brain imaging performed within 72 hours. Changes in ICP may have occurred in between ONSD ultrasound and brain imaging, reducing correlation between these 2 scanning modalities. The number of participants for whom radiological changes consistent with raised ICP were noted on baseline brain imaging was small, and likely why ONSD did not significantly correlate with brain imaging labeled as “raised ICP.” We were unable to correlate ONSD with invasively measured ICP.

Finally, it is not known if participants received corticosteroids. Participants in this study were enrolled into 1 of 2 randomized, double blind, placebo-controlled trials of adjunctive corticosteroid therapy in TBM, and the dexamethasone/placebo allocation remains unknown. Baseline data including baseline ONSD ultrasound and 3-dimensional brain imaging, prior to dexamethasone or placebo administration, were unaffected by this. Neurological complications and death by 3 months may be affected by dexamethasone; an improving individual patient ONSD trend may reflect dexamethasone use if ICP was raised due to a dexamethasone-responsive cause such as neuroinflammation. However, this blinded allocation should not affect the ability of ONSD to monitor and chart this trend.

A strength of our study is that study data were collected as part of 2 clinical trials with study protocols, standard operating procedures, and careful conduct of research. Ours is the largest study to date of ONSD ultrasound in TBM, combining longitudinal ONSD data in individual participants with clinical endpoints. Brain imaging was independently reported by a neuroradiologist and correlated with CSF parameters, which reflect measurements of inflammation at the site of disease rather than correlation with blood parameters.

In conclusion, we demonstrated that higher ONSD values were associated with increased disease severity, brain-imaging abnormalities, and an increased risk of death by 3 months. Optic nerve sheath diameter ultrasound has potential for use as a bedside tool for ICP monitoring in TBM and identifying those at greatest risk of neurological complications and death.

## Supplementary Data

Supplementary materials are available at *Clinical Infectious Diseases* online. Consisting of data provided by the authors to benefit the reader, the posted materials are not copyedited and are the sole responsibility of the authors, so questions or comments should be addressed to the corresponding author.

ciaa1823_suppl_Supplementary_Material

ciaa1823_suppl_SupplementaryFigure1

## Notes

***Acknowledgments.*** The authors thank the patients who participated, the doctors and nurses who cared for the patients, and the laboratory staff at the Hospital for Tropical Diseases.

***Financial support.*** This work was supported by a Wellcome Trust UK Investigator Award (grant number 110179/Z/15/Z; to G. E. T.) and by a Wellcome Trust UK Innovation for Impact in Low- and Middle-Income Countries Award (grant number WT217650/Z/19/Z).

***Potential conflicts of interest.*** The authors: No reported conflicts of interest. All authors have submitted the ICMJE Form for Disclosure of Potential Conflicts of Interest.
