# Supplementary material for: Optic Nerve Sheath Ultrasound for the Detection and Monitoring of Raised Intracranial Pressure in Tuberculous Meningitis
Source: Clin Infect Dis. 2020 Dec 7;73(9):e3536–44. doi: 10.1093/cid/ciaa1823 (PMC8563195; doi:10.1093/cid/ciaa1823)
Supplement: ciaa1823_suppl_Supplementary_Material [file ciaa1823_suppl_supplementary_material.docx]

**Supplementary appendix**

**Vietnam ICU Translational Applications Laboratory (VITAL) investigators:**

**OUCRU inclusive authorship list in Vietnam** (alphabetic order by surname): Dang Trung Kien, Dong Huu Khanh Trinh, Joseph Donovan, Du Hong Duc, Ronald Geskus, Ho Bich Hai, Ho Quang Chanh, Ho Van Hien, Hoang Minh Tu Van, Huynh Trung Trieu, Evelyne Kestelyn, Lam Minh Yen, Le Nguyen Thanh Nhan, Luu Phuoc An, Nguyen Lam Vuong, Nguyen Than Ha Quyen, Nguyen Thi Le Thanh, Nguyen Thi Phuong Dung, Ninh Thi Thanh Van, Phan Nguyen Quoc Khanh, Phung Khanh Lam, Phung Tran Huy Nhat, Guy Thwaites, Louise Thwaites, Tran Minh Duc, Trinh Manh Hung, Hugo Turner, Jennifer Ilo Van Nuil, Sophie Yacoub

**Hospital for Tropical Diseases, Ho Chi Minh City** (alphabetic order by surname): Cao Thi Tam, Duong Bich Thuy, Ha Thi Hai Duong, Ho Dang Trung Nghia, Le Buu Chau, Luong Thi Hue Tai, Nguyen Hoan Phu, Nguyen Quoc Viet, Nguyen Thanh Nguyen, Nguyen Thanh Phong, Nguyen Thi Kim Anh, Nguyen Van Hao, Nguyen Van Thanh Duoc, Nguyen Van Vinh Chau, Pham Kieu Nguyet Oanh, Phan Tu Qui, Phan Vinh Tho

**University of Oxford** (alphabetic order by surname): David Clifton, Mike English, Heloise Greeff, Huiqi Lu, Jacob McKnight, Chris Paton

**Imperial College London** (alphabetic order by surname): Pantellis Georgiou, Bernard Hernandez Perez, Kerri Hill-Cawthorne, Alison Holmes, Stefan Karolcik, Damien Ming, Nicolas Moser, Jesus Rodriguez Manzano

**King’s College London** (alphabetic order by surname): Alberto Gomez, Hamideh Kerdegari, Marc Modat, Reza Razavi

**ETH Zurich** (alphabetic order by surname): Abhilash Guru Dutt, Walter Karlen, Michaela Verling, Elias Wicki

**The University of Melbourne** (alphabetic order by surname):

Linda Denehy, Thomas Rollinson

**Supplementary table 1: MRI and CT brain reporting template**

| **Study identification number** |  | ***Enter no.* ______** |
| --- | --- | --- |
| **Quality OK?** |  | ***Yes / No*** |
| **MRI?** |  | ***Yes / No*** |
| **CT?** |  | ***Yes / No*** |
| **Normal?** |  | ***Yes / No*** |
| **Evidence of bleeding?** |  | ***Yes / No*** |
|  | | |
| **Meningeal enhancement?** |  | ***Yes / No*** |
| *If yes* | Basal | ***Yes / No*** |
|  | Sylvian | ***Yes / No*** |
|  | Convexity | ***Yes / No*** |
|  | Posterior fossa | ***Yes / No*** |
|  | Ependymal | ***Yes / No*** |
|  | | |
| **Hydrocephalus?** |  | ***Yes / No*** |
| *If yes* | Communicating | ***Yes / No*** |
|  | | |
| **Infarcts?** |  | ***Yes / No*** |
| *If yes* | Total number | ***Enter no.* ______** |
|  | No. diffusion restricted | ***Enter no.* ______** |
|  | No. cortical | ***Enter no.* ______** |
|  | No. callosal | ***Enter no.* ______** |
|  | No. lacunar | ***Enter no.* ______** |
|  | | |
| **Tuberculomas?** |  | ***Yes / No*** |
| *If yes* | Total no. | ***Enter no.* ______** |
|  | No. parenchymal | ***Enter no.* ______** |
|  | No. ependymal | ***Enter no.* ______** |
|  | No. meningeal | ***Enter no.* ______** |
|  | | |
| **Local sulcal effacement?** |  | ***Yes / No*** |
| **Hemispheric sulcal effacement?** |  | ***Yes / No*** |
| **Basal cistern effacement?** |  | ***Yes / No*** |
| **Consistent with elevated ICP?** |  | ***Yes / No*** |
| **Additional notes?** |  | |

CT=Computed tomography. ICP=Intracranial pressure. ID=Identification. MRI=Magnetic resonance imaging.

**Supplementary table 2: Study drug doses in ACT HIV and LAST ACT after randomisation**[21,22]

|  | **MRC Grade I**  Daily dexamethasone dose/route | **MRC Grades II and III**  Daily dexamethasone dose/route |
| --- | --- | --- |
| Week 1 | 0.3 mg/kg/24 hrs IV | 0.4 mg/kg/24 hrs IV |
| Week 2 | 0.2 mg/kg/24 hrs IV | 0.3 mg/kg/24 hrs IV |
| Week 3 | 0.1 mg/kg/24 hrs IV | 0.2 mg/kg/24 hrs IV |
| Week 4 | 3mg/24 hrs oral | 0.1 mg/kg/24 hrs IV |
| Week 5 | 2mg/24 hrs oral | 4 mg/24 hrs oral |
| Week 6 | 1 mg/24 hrs oral | 3 mg/24 hrs oral |
| Week 7 | Stop | 2 mg/24 hrs oral |
| Week 8 |  | 1 mg/24 hrs oral |

Kg=Kilogram. IV=Intravenous. MRC=Medical Research Council.

**Supplementary table 3: Correlation of baseline ONSD with baseline disease severity and CSF inflammatory parameters**

| **Parameter** | **Total No.** | **Correlation co-efficient** | **P value** |
| --- | --- | --- | --- |
| Highest temperature (^0^C) | 71 | 0.08 | 0.49 |
| Serum sodium (mmol/L) | 65 | -0.09 | 0.48 |
| Lumbar CSF opening pressure (cmH_2_0) | 49 | 0.09 | 0.56 |
| CSF WBC (cells/mm^3^) | 72 | 0.08 | 0.52 |
| CSF neutrophils (%) | 71 | 0.23 | 0.05 |
| CSF neutrophil count (cells/mm^3^) | 71 | 0.15 | 0.22 |
| CSF/blood glucose ratio | 71 | -0.05 | 0.65 |
| CSF protein (g/L) | 72 | 0.11 | 0.38 |
| CSF lactate (mmol/L) | 72 | 0.12 | 0.31 |

Co-efficient and significance (p value) are shown using Spearman’s rank correlation co-efficient.
CI=Confidence interval. CSF=Cerebrospinal fluid. ONSD=Optic nerve sheath diameter. WBC=White blood cells.

**Supplementary figure 1: ROC curve plotting true positive rate (sensitivity) and false**

**positive rate (1-specificity) for ONSD as a predictor of death by 3 months**


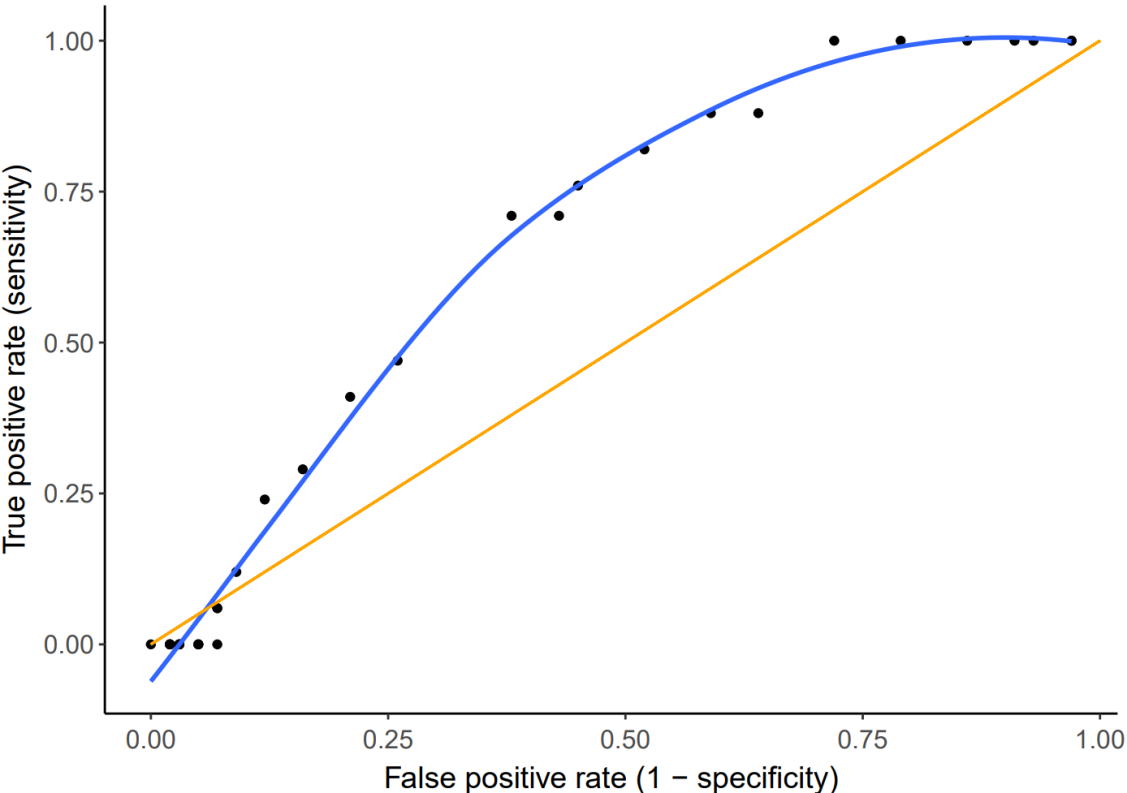


ONSD=Optic nerve sheath diameter. ROC=Receiver operating characteristic

**Supplementary material 1**

Diagnostic criteria for TBM [24]

|  |  | | Diagnostic score |
| --- | --- | --- | --- |
| **Clinical criteria** | (Maximum category score=6) | | |
|  | Symptom duration of >5 days | | 4 |
|  | Systemic symptoms suggestive of tuberculosis (one or more of the following): weight loss (or poor weight gain in children), night sweats, or persistent cough for >2 weeks | | 2 |
|  | History of recent (within past year) close contact with an individual with pulmonary tuberculosis or a positive TST or IGRA (only in children <10 years of age) | | 2 |
|  | Focal neurological deficit (excluding cranial nerve palsies) | | 1 |
|  | Cranial nerve palsy | | 1 |
|  | Altered consciousness | | 1 |
| **CSF criteria** | (Maximum category score=4) | | |
|  | Clear appearance | | 1 |
|  | Cells: 10-500 per μl | | 1 |
|  | Lymphocytic predominance (>50%) | | 1 |
|  | Protein concentration >1 g/L | | 1 |
|  | CSF to plasma glucose ratio of less than 50% or an absolute CSF glucose concentration less than 2.2mmol/L | | 1 |
| **Cerebral imaging criteria** | (Maximum category score=6) | | |
|  | Hydrocephalus | | 1 |
|  | Basal meningeal enhancement | | 2 |
|  | Tuberculoma | | 2 |
|  | Infarct | | 1 |
|  | Pre-contrast basal hyperdensity | | 2 |
| **Evidence of tuberculosis elsewhere** | (Maximum category score=4) | | |
|  | Chest radiograph suggestive of active tuberculosis: signs of tuberculosis=2; miliary tuberculosis=4 | | 2 or 4 |
|  | CT/ MRI/ ultrasound evidence for tuberculosis outside the CNS | | 2 |
|  | AFB identified or *Mycobacterium tuberculosis* cultured from another source-i.e., sputum, lymph node, gastric washing, urine, blood culture | | 4 |
|  | Positive commercial *M. tuberculosis* NAAT from extra-neural specimen | | 4 |
| **Diagnostic criteria based on total score:**  Possible TBM: score 6-9 (if no brain imaging) or 6-11 (if brain imaging)  Probable TBM: score >9 (if no brain imaging) or >11 (if brain imaging)  Definite TBM: acid-fast bacilli seen in CSF or *M. tuberculosis* cultured or detected by commercial NAAT in CSF | |  |  |

**Supplementary material 2**

Guidelines for treatment of tuberculosis [21,22]

First line treatment

Rifampicin (10mg/kg/24 hrs; maximum 600mg), isoniazid (5mg/kg/24hrs; maximum 300mg), pyrazinamide (25mg/kg/24hrs; maximum 2g) and ethambutol (20mg/kg/24 hrs; maximum 1.2g) are given for for at least the first 2 months of treatment.

Pyrazinamde will then be stopped and rifampicin, isoniazid and ethambutol (at the same doses) will then be given until at least 12 months anti-tuberculosis treatment in total has been given. If pyrazinamide cannot be given for at least 2 months, for example to to drug side effects, then total treatment should be at least 12 months.

Isoniazid-resistant tuberculosis

Option 1: Follow the standard regimen above, but replace isoniazid with levofloxacin (20mg/kg/24 hrs; maximum 1000 mg/day). Pyrazinamide can be used throughout treatment in those with more severe disease who are responding slowly.

Option 2: Stop isoniazid and treat with rifampicin, ethambutol and pyrazinamide for the entire 9-12 months of treatment. This option is not suitable for those with confirmed ethambutol resistant bacteria; these participants should be treated with option 1.

Multi-drug resistant tuberculosis

Second line treatment for MDR TBM should be given as soon as possible, following National guidelines and local policies.
