# Supplementary figures and images for: Optic Nerve Sheath Ultrasound for the Detection and Monitoring of Raised Intracranial Pressure in Tuberculous Meningitis
Source: Clin Infect Dis. 2020 Dec 7;73(9):e3536–44. doi: 10.1093/cid/ciaa1823 (PMC8563195; doi:10.1093/cid/ciaa1823)

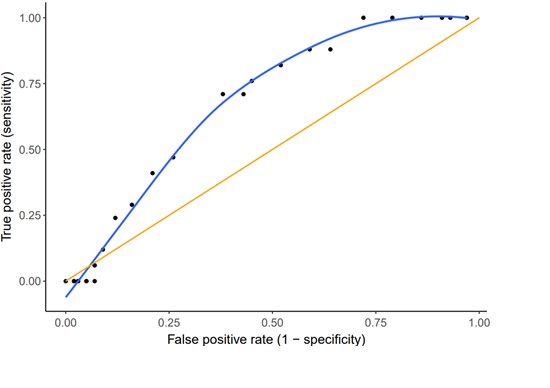

Supplement: ciaa1823_suppl_SupplementaryFigure1 [file ciaa1823_suppl_supplementaryfigure1.jpeg]
